# Studies of Interfacial Interaction between Polymer Components on Helical Nanofiber Formation via Co-Electrospinning

**DOI:** 10.3390/polym10020119

**Published:** 2018-01-26

**Authors:** Huihui Wu, Shihang Zhao, Wenhua Ding, Lei Han

**Affiliations:** 1 Pan Tianshou Arts and Design Academy, Ningbo University, Ningbo 315211, China; 2 School of Materials Science & Chemical Engineering, Ningbo University, Ningbo 315211, China; 17815936324@163.com (S.Z.); hanlei@nbu.edu.cn (L.H.); 3 Wu Han Aplus Technology Co., Ltd., Wuhan 430000, China; 18703603920@163.com

**Keywords:** interfacial interaction, polymer components, helical nanofiber, co-electrospinning

## Abstract

Helical fibers in nanoscale have been of increasing interest due to their unique characteristics. To explore the effect of polymer type on helical fiber formation, three polymer systems, Poly(*m*-phenylene isophthalamide) (Nomex)/polyurethane (TPU), polystyrene (PS)/TPU and polyacrylonitril (PAN)/TPU are used to fabricate helical nanofibers via co-electrospinning. Differential scanning calorimetry (DSC), Fourier transform infrared spectroscopy (FTIR) and Zeta potential were employed to investigate the interfacial interaction between the two phases of the polymer system. The larger rigidity differential of Nomex and TPU leads to a larger interfacial interaction. The hydrogen bonds help to increase the interfacial interaction between Nomex and TPU components. The attractive force between the chloride-ions contained in Nomex molecules and the free charges on the solution surface lead to a longitudinal interfacial interaction in the Nomex/TPU system. The analysis results provide the explanation of the experimental results that the Nomex/TPU system has the greatest potential for producing helical nanofibers, while the PS/TPU and PAN/TPU systems cannot fabricate helical fibers effectively. This study based on the interfacial interaction between polymer components provides an insight into the mechanism of helical fiber formation.

## 1. Introduction

The existence of helical structures in nature, such as in tendrils and in fine wool, is the consequence of differential growth, which develops because of asymmetric distribution of the predominant cell types. This may generate different extensions or shrinkages of the internal fiber regions [1,2]. The exploration of applying this mechanism to prepare biomimetic materials by coupling helical structures with micro/nanofibers has been of great interest due to the potential applications of the micro/nanoscale helical fibers [3,4]. Since springs are a kind of unique construction element allowing large elastic deformation, helical fibers in the micro/nanoscale resembling springs may act as a mechanical device operating in micro-systems. Moreover, the three-dimensional structure of the helices may lead to a larger porosity and surface area of the micro/nanofiber mat, and therefore provide new materials for filtration and absorbtion.

Electrospinning is a single-step process to produce nanofibers, opening possibilities for preparing helical fibers in the micro/nanoscale. In earlier studies, several researchers reported the fabrication of helical fibers based on a conventional electrospinning system from one or two-component solutions [5,6,7,8]. Unfortunately, the helical structures obtained were observed as two-dimensional, and the formed helix diameters were up to several dozens of micrometers. Meanwhile, their explanations for the formation mechanism of the helical structure were not so convincing. The formation of these helical fibers was finally regarded as the result of jet buckling during whipping and hitting with the collector surface [9].

Co-electrospinning, which is characterized by its specially-designed, multi-channel spinneret, is a simple and efficient way for fabricating composite nanofibers. With the aid of the co-electrospinning technique, several researchers successfully prepared three-dimensional helical nanofibers from two-component solutions. Lin et al. [10] obtained nanoscale biomimetic wool fibers by electrospinning Polyacrylonitrile (PAN) and Polyurethane (PU) using a side-by-side co-electrospinning arrangement. Chen et al. [11,12] utilized three kinds of co-electrospinning spinnerets to produce nanosprings from PU and Nomex. Using side-by-side electrospinning, Zhang et al. [13] reported the generation of fibers with curled and helical morphologies from poly(ethylene glycol terephthalate) (HSPET) and poly(ethylene propanediol terephthalate) (PTT). In the above research, the authors attributed the generation of helical fibers to the fact that the two components involved in co-electrospinning display different shrinkages after electrospinning. The mechanism for fiber curvature is also provided by the asymmetric deformation of tendrils and fine wool.

Based on the concept that the parallel arrangement of an elastomeric and a stiff polymer in co-electrospinning may introduce longitudinal stress and result in coiled shapes of the bicomponent fibers, our previous studies [14,15] reported the fabrication of helical nanofibers via co-electrospinning. Also reported in the studies were the effects of some parameters, including electric field, material parameters, solution parameters and processing parameters, on the formation of helical nanofibers. In co-electrospinning, additional phases introduce an interfacial interaction, which we believe plays a key role in the formation of helical structures. In this present work, three component systems, which represent three kinds of polymer composition arrangements in co-electrospinning, were chosen to be studied. We focused on the interfacial interaction between two components induced by different polymer structures and intrinsic properties, including polymer chain rigidity, miscibility and hydrogen bonding of the two solutions, and Couloumb force between the molecules and the solution surface. Thermal and spectroscopic techniques including DSC, FTIR and Zeta potential were utilized to study the interaction behavior of the polymer pairs. This study provides insight into the helical fiber formation, and the guide for choosing polymers when producing helical nanofibers via co-electrospinning.

## 2. Results

**Figure 1:**
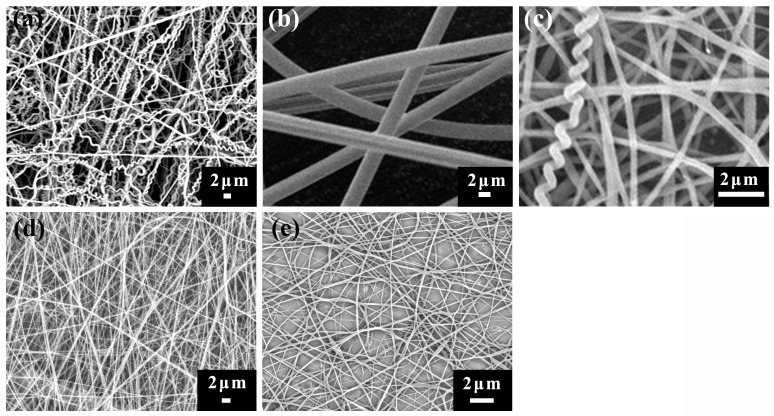
SEM images of three component systems: (**a**) Nomex/TPU fibers from 12 wt % Nomex with 1.8 wt % LiCl and 18 wt % TPU, (**b**) PS/TPU fibers from 20 wt % PS and 18 wt % TPU, (**c**) PAN/TPU fibers from 12 wt % PAN and 18 wt % TPU, (**d**) 12 wt % Nomex with 1.8 wt % LiCl and (**e**) 18 wt % TPU.

Figure 1a–c shows the results of the co-electrospinning experiments with three component systems under the processing conditions of 20 kV applied voltage, 15 cm working distance, and 0.15 mL/h flow rate for both component solutions. The scanning electron microscope (SEM) images show that a considerable amount of helical fibers with 100–500 nm fiber diameters and 500–800 nm helix diameters were observed in the Nomex/TPU fiber web, indicating that helical nanofibers can be fabricated effectively with the Nomex/TPU system. In contrast, the fabrication of helical fibers is much less effective with the PAN/TPU and PS/TPU systems. Only a few fibers show helical structures in the PAN/TPU fiber web, while only straight fibers are formed when applying the PS/TPU system. Although Figure 1 shows the results under the same processing conditions, we tried various processing conditions for the three component systems, and the experiments show similar results, namely that the Nomex/TPU fibers can generate helical structures more effectively compared with the other two component systems. These experiments demonstrate that polymer type plays a crucial role in the generation of helical fibers. As a comparison, Figure 1d–e shows the results of the single electrospinning experiments of pure Nomex and pure TPU under the same processing conditions as the co-electrospinning experiments. As we can see, there are no helical fibers formed when conducting the pure single electrospinning. This removes the influence of synthesis parameters and external electrospinning factors on the formation of helical fibers, and proves that an interfacial interaction between Nomex/TPU components does exist.

## 3. Discussion

In this study, the fabrication of helical nanofibers is based on the combination of an elastomeric with a stiff component in co-electrospinning. In our experiments, TPU is used as the elastomeric component, while Nomex, PAN and PS are chosen as the stiff part. An interfacial interaction is introduced between the two components due to the different mechanical properties they display. In the following, focusing on the interfacial interaction induced by polymer structure and intrinsic properties, we try to explore the effect of polymer type on the formation of helical fibers.

### 3.1. Rigidity of Polymer Chain

In the co-electrospinning process, the longitudinal compressive stress arising from the resilience of the flexible component (i.e., TPU) and the rigidity of the stiff component (i.e., Nomex, PS and PAN) is fundamental for the formation of helical structures. It can be predicted that the more significant the rigidity (or flexibility) differential of the two components, the greater the potential for the component system to generate helical structures in co-electrospinning due to the greater interfacial stress between the components.

**Figure 2:**
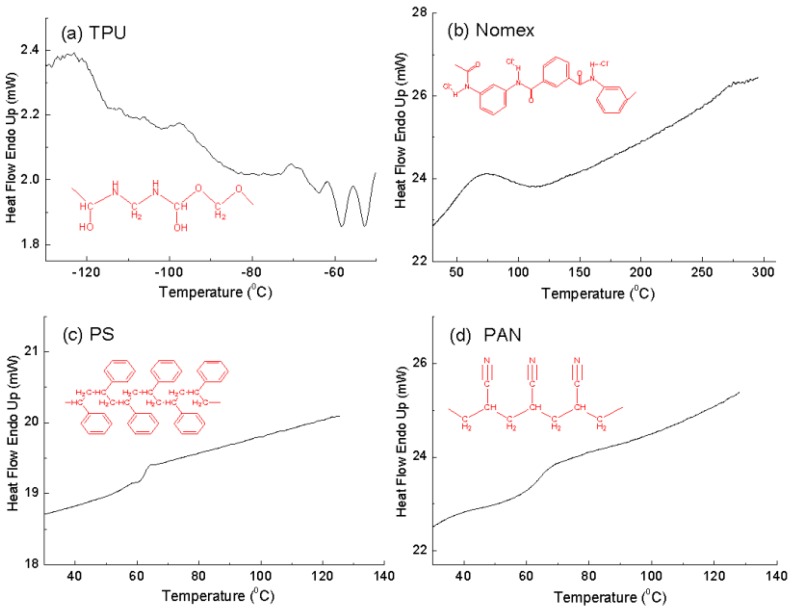
Differential scanning calorimetry (DSC) thermograms and molecular formulas of the four polymer components: (**a**) TPU, (**b**) Nomex, (**c**) PS, and (**d**) PAN.

The glass transition temperature of a polymer, *T*g, is an important intrinsic property that influences both the physical and mechanical properties including strength, toughness and stiffness. Typically, polymers with high chain rigidity have higher *T*g [16,17]. DSC analysis is one of the convenient methods for determining the glass transition temperature of the polymer. Figure 2 shows the DSC thermograms of the components used in the study, TPU, Nomex, PS and PAN. As is known, TPU has a *T*g of about −85 °C, indicating a quite flexible polymer chain (Figure 2a). We turned our attention to the three stiff components shown in Figure 2b–d. Nomex has a higher *T*g (about 110 °C) than PS and PAN (about 70 °C), indicating the greater chain rigidity of Nomex. Also shown in Figure 2 are the molecular formulas of the polymers. It can be seen that the stiff chain backbone arising from the benzene ring groups contributed to the rigidity of Nomex, while the flexible backbone contributed to the flexibility of PAN. As to the chain of PS, although it contains stiff side groups (benzene ring groups), the flexible backbone determines the lesser rigidity of PS. The larger chain rigidity differential of the Nomex and TPU leads to a larger interfacial interaction between them, and consequently provides a greater potential for the Nomex/TPU component system to produce helical fibers in co-electrospinning, as shown in the experimental results.

### 3.2. Miscibility Behavior and Hydrogen Bonding in Blends

Co-electrospinning involves two polymer solutions which are blended in the outlet of the spinneret. When stretched by the electric field, the interfacial tension occurs between the two components. Immiscible blends may result in the failure of the fabrication of bicomponent fibers due to less interaction between the two components. At the opposite extreme of the immiscible case is the completely miscible case, where the interfacial interaction may vanish in a one-phase system of the completely miscible blend. Helical fibers can hardly be fabricated in these two cases. It is expected that the extent of miscibility in blends should be properly arranged to fabricate helical fibers effectively. To obtain polymer blends with good miscibility, it is usually necessary to ensure that hydrogen bonding exists between two base components. In this study, FTIR and DSC are used to analyze the miscibility behavior and hydrogen bonding in the blends of Nomex/TPU, PS/TPU and PAN/TPU systems. Blends with different component pairs were prepared by solution blending. Blend solutions were stirred for 6–8 h, and were allowed to evaporate slowly at 50 °C for one day. Films of the blends were then dried at 80 °C for two days to ensure the total elimination of solvents.

**Figure 3:**
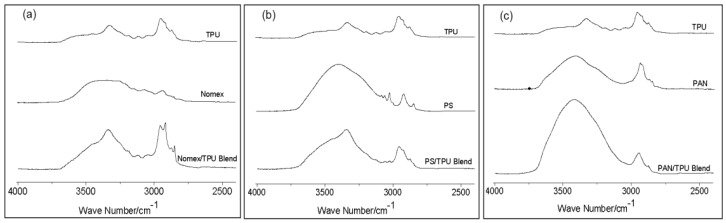
Fourier transform infrared spectroscopy (FTIR) of the three component systems including pure polymers and the blends: (**a**) Nomex/TPU, (**b**) PS/TPU, and (**c**) PAN/TPU.

Figure 3 shows infrared spectra in the range of 2500–4000 cm^−1^ of the three systems. Figure 3a shows a broad band centered at 3306 cm^−1^ for pure TPU and Nomex, corresponding to the hydrogen-bonded –NH. Although the broad band of the Nomex/TPU blend keeps at 3306 cm^−1^, the intensity of the –NH group increases, indicated the formation of a new hydrogen bonding between the –NH in TPU and the oxygen in Nomex. As for the case of the PS/TPU system shown in Figure 3b, a broad band, corresponding the –CH group, centered at 3400 cm^−1^ for pure PS. In the PS/TPU blend, the position of this band shifts to a slightly lower wavenumber, which is closer to pure TPU. Meanwhile, the intensity of the band for the PS/TPU blend is in the range between the intensities for the two pure components, suggesting that there is no evidence of hydrogen bonding between PS and TPU. We turned our attention to the PAN/TPU system. Figure 3c shows a band with significantly increased intensity centered at 3418 cm^−1^ for the PAN/TPU blend. This phenomenon can be attributed to the hydrogen-bonding interactions between the –NH in TPU and the nitrogen in PAN. These data from Figure 3 suggest that the extent of miscibility is different for the three component systems. Nomex is partially miscible with TPU due to the formation of hydrogen bonding between their polymer chains. PS and TPU are immiscible due to the absence of the specific intermolecular interaction. PAN is completely miscible with TPU and the blend becomes a one-phase system due to strong hydrogen bonding between their chains.

**Figure 4:**
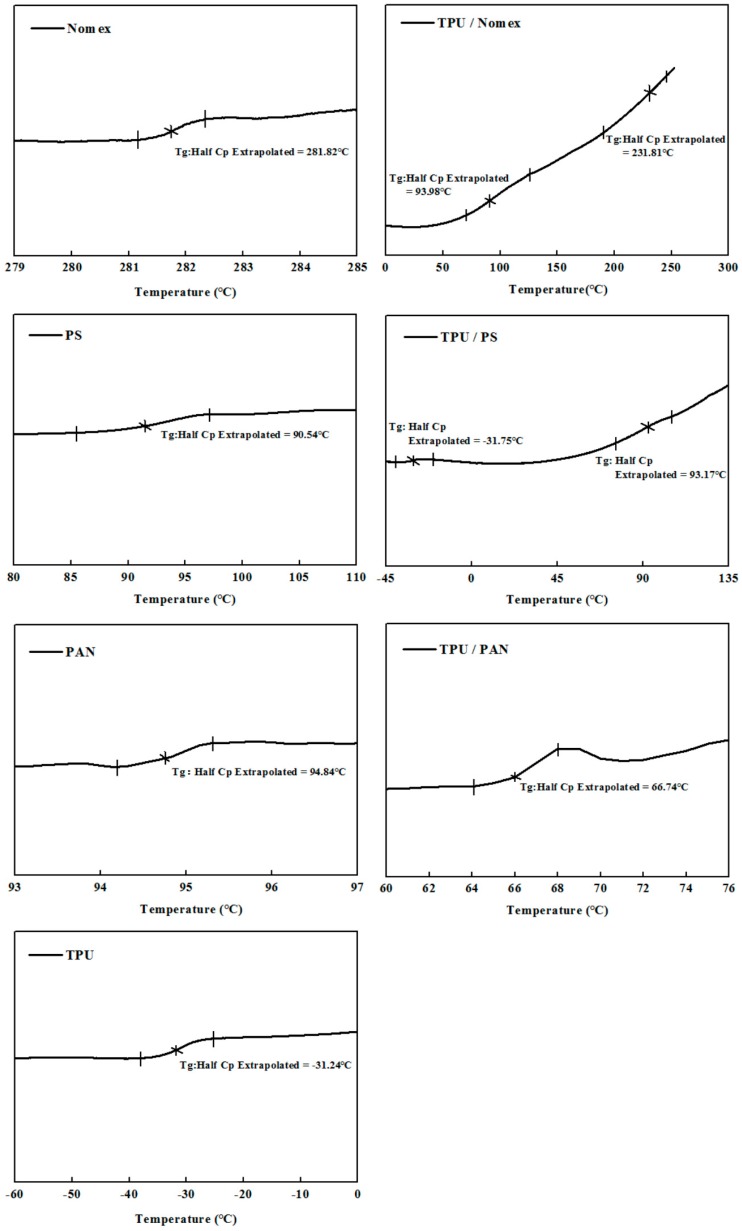
DSC thermograms of the three component systems including the pure polymers and the blends.

The miscibility of the polymer blends can be further analyzed by the DSC technique. If the two components are completely incompatible with each other, then they have two *T*gs that correspond to the two components. If the two components are partially compatible, then the blends have two *T*gs shifted towards each other when the compositions of blends are out of the range of compatibility. If the components in binary blends are fully compatible, then the blends display a single *T*g within the full range of compositions [18]. Figure 4 shows the DSC thermograms of the Nomex/TPU, PS/TPU and PAN/TPU systems. Here we need to emphasize that the components employed in this analysis were prepared by TPU, Nomex, PS and PAN solutions, rather than the chopped fibers or pellets used in the DSC analysis for polymer chain rigidity (Figure 2). Figure 4 illustrates that in the Nomex/TPU blend, there are two *T*gs (93.98 and 231.81 °C) located between the *T*gs of the two individual polymers (−31.24 °C for pure TPU and 281.82 °C for pure Nomex), which gives an indication of partial miscibility in the blend. Although there are two *T*gs shown in the PS/TPU blend, unlike the partial miscibility observed in Figure 4a, the *T*gs (−31.75 and 93.17 °C) of the blend are out of the range of those of pure TPU and pure PS (−31.24 and 90.54 °C). This phenomenon indicates an immiscibility behavior of the PS/TPU blend. As can be seen in the PAN/TPU blend curve, a single *T*g (66.74 °C) strongly suggests that the PAN/TPU is fully miscible and exist as a one-phase system.

By analyzing the miscibility of the three systems, we believe that the partially miscible Nomex/TPU system tends to generate helical structures due to the intensified interfacial interaction attributed to hydrogen bonding. The PS/TPU system can hardly form helical fibers in coelectrsopinning because when there is no compatibility between the two blends, the interfacial interaction is too weak to support the helical interfacial stress, while if the two polymers are fully compatible, like the PAN/TPU system, the rigidity differential of the two components disappears, which also makes it difficult to meet the helical fiber formation conditions. This analysis coincides with the experimental results.

### 3.3. Coulumb Force between Molecules and Solution Surface

**Figure 5:**
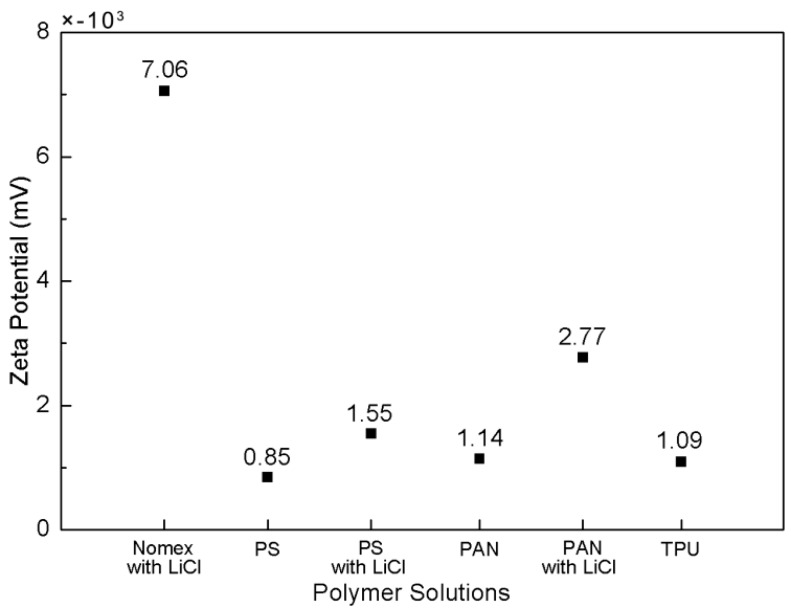
Zeta potential of different polymer solutions.

When preparing the polymer solutions for co-electrospinning, we found that PS, PAN and TPU can be dissolved in the pure solvents DMF, DMAc and DMF/THF, respectively. However, Nomex is difficult to dissolve in pure solvents due to its highly structured molecular arrangement. The dissolution of Nomex in DMAc/LiCl, the mechanism of which was introduced in our previous study [15], leads to the combination of the negatively charged chloride ions with the polymer chain of Nomex. In this study, we used Zeta potential analyzer for the quantification of the magnitude of the charge contained within the polymer molecules. Figure 5 shows the obtained Zeta potentials (negative) for the Nomex/DMAc with LiCl, PS/DMF, PAN/DMAc, and TPU/(DMF/THF) solutions. For comparison, the Zeta potentials of the PS and PAN solutions with LiCl are also measured and shown in the figure. The amount of LiCl used in the three types of solutions are all 1.8 wt % in order to exclude the influence of the solution conductivity. It can be seen that the Zeta potential for the PAN, PS and TPU (without LiCl) are very low, indicating that few free charges are contained in the molecules. In contrast, the Zeta potential for Nomex is much higher. Although the Zeta potentials increase a bit for the PS and PAN solutions when adding LiCl, they are still much lower than that of the Nomex solution. The small magnitude of Zeta potential for the PS and PAN solutions with LiCl indicate that the chloride ions are dispersed in the solutions, rather than accumulated in the polymer chain. On the contrary, the great magnitude of Zeta potential for the Nomex solution indicates that the negative charges are contained in the molecules as expected.

**Figure 6:**
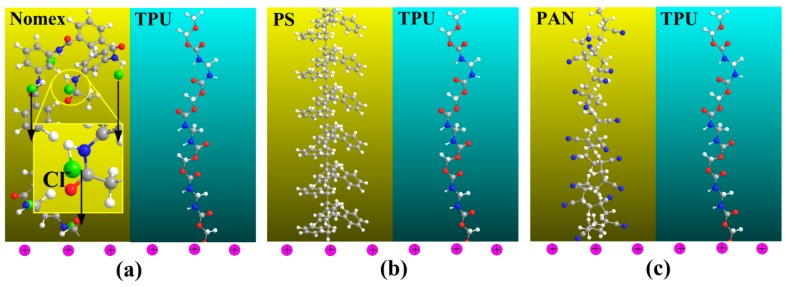
Schematic illustration of attraction force exerted on three polymer systems (**a**) Nomex/TPU, (**b**) PS/TPU, and (**c**) PAN/TPU [15].

When the polymer solutions are charged by the applied high voltage, the free charges are accumulated on the surface of the solution. It can be predicted that in the Nomex solution, there will be a Coulomb force of attraction generated between the positive charges at the solution surface and the negative charges carried by chloride ions in the polymer chain. In contrast, due to the lack of free charges contained in the molecules, the situation is different for the other three components. In other words, the Coulomb force between the charges on the solution surface and within the molecules will not generate for the TPU, PS and PAN solutions. A schematic illustration of the composite solutions for the three component systems being charged is shown in Figure 6 [15]. It can be seen that the Coulomb force for the Nomex solution generates a longitudinal interfacial stress between the Nomex and TPU solutions, which helps to form helical structures (Figure 6a). Figure 6b,c illustrates that the interfacial interaction arising from the Coulomb force cannot be observed for the PS/TPU and PAN/TPU systems.

From the above analyses of the three aspects, it can be summed up that among the three component systems, the Nomex/TPU system has the most potential to produce helical nanofibers via co-electrospinning, which is consistent with the experiments.

## 4. Materials and Methods

### 4.1. Materials

Poly(*m*-phenylene isophthalamide) (Nomex) chopped fibers and polyacrylonitrile (PAN) chopped fibers was purchased from Shanghai Xiangrun Trading Co., Ltd., Shanghai, China. Polystyrene (PS) (*M*_w_ = 350,000 g/mol) was purchased from Sigma-Aldrich Co., Ltd., Darmstadt, Germany. Thermoplastic polyurethane (TPU, Desmopan DP 2590A) was from Bayer Material Science (Shanghai) Co., Ltd., Shanghai, China. *N*,*N*,-dimethylacetamide (DMAc) (0.938–0.942 g/mL at 20 °C), *N*,*N*-dimethylformamide (DMF) (0.945–0.950 g/mL at 20 °C), Tetrahydrofuran (THF) (0.887–0.889 g/mL at 20 °C), and Lithium chloride anhydrous (LiCl) (*M*_w_ = 42.39 g/mol) were purchased from Shanghai Chemical Reagents Co., Ltd., Shanghai, China. All of these materials were used without further purification.

### 4.2. Co-Electrospinning

**Figure 7:**
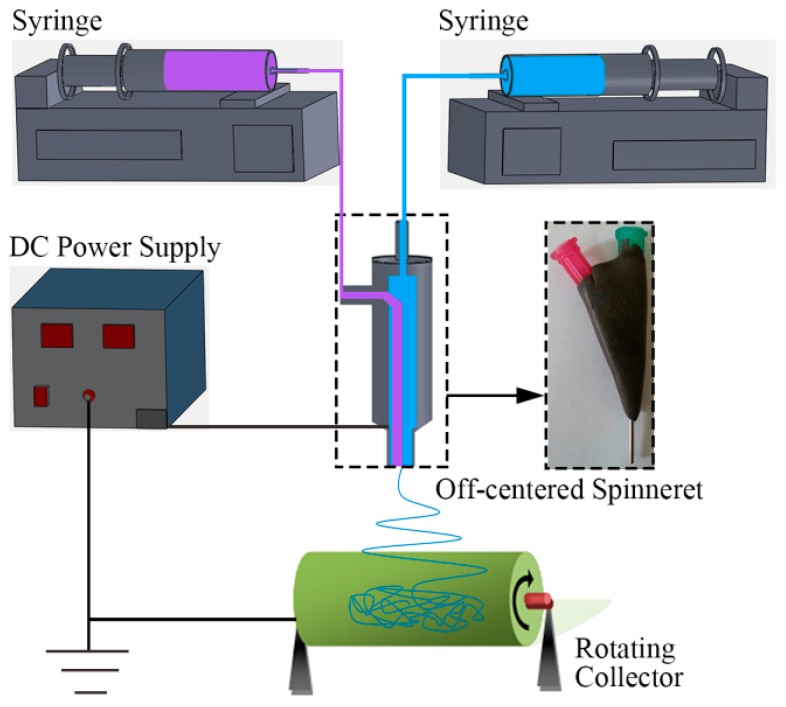
Schematic of the off-centered co-electrospinning system [14].

Three component systems, Nomex/TPU, PS/TPU, and PAN/TPU were chosen for co-electrospinning. Nomex solution with 12 wt % concentration was prepared by dissolving Nomex chopped fibers in the mixture of DMAc with 1.8 wt % LiCl, stirred for 24 h at 100 °C. PS solution with 20 wt % concentration was prepared by dissolving PS pellets in DMF, stirred for 5 h at ambient temperature. PAN solution with 12 wt % concentration was prepared by dissolving PAN fibers in DMAc, stirred for 5 h at ambient temperature. TPU solution with 18 wt % concentration was prepared by dissolving TPU pellets in mixture solvents of DMF/THF (3/1 volume ratio), stirred for 5 h at ambient temperature. As shown in Figure 7, a co-electrospinning system was used to eject polymer solutions through an off-centered spinneret, which was detailed in our previous work [14]. The solutions for the core and shell solutions were separately fed into the spinneret via corresponding syringes and pumps (KDS 220, KD Scientific, Inc., Ringoes, NJ, USA). A high voltage supply (ES-60P 10W/DDPM, Gamma High Voltage Research, Co., Ltd., Ormond Beach, FL, USA) was applied to the spinneret and the rotating cylinder (linear velocity of about 14.24 cm/s). All experiments were performed at about 25 °C in air at 40–60% relative humidity.

### 4.3. Characterization

*Fiber morphology.* The morphology of the resultant core-shell fibers were observed under a scanning electron microscope (SEM) (JSM-5600LV, JEOL Ltd., Tokyo, Japan) after gold coating (coating time is 60 s). The average fiber diameter was calculated from the SEM images using Photoshop CS 6 (Adobe System Inc., San Jose, CA, USA) software from a collection of 200 fibers.

*DSC*. The glass transition temperatures of the blends were implemented using a DSC machine (DSC 4000, PerkinElmer Ltd., Billerica, MA, USA) in a nitrogen atmosphere with temperature range −80~350 °C. The weight of samples, which were placed in a DSC sample cell, was 5 to 10 mg. The samples were heated to 350 °C at 10 °C/min and held at 350 °C for 5 min to eliminate any thermal history. The samples were then quenched to −80 °C and finally reheated to 350 °C at a rate of 10 °C/min. The reheating scans were recorded.

*FTIR*. Infrared spectra were recorded on a FTIR spectrophotometer (Bruker Vector 33, Bruker Ltd., Rheinstetten, Germany). Infrared spectra of polymer blend films were determined by using the conventional KBr disk method. The solution containing the blends were cast onto KBr disk and dried under at room temperature for two days. The film used in this study was sufficiently thin to obey the Beer-Lambert law.

*Zeta potential*. The surface charge of the polymer chains dispersed in DMF at a concentration of 5 mg/mL was measured using a zeta potential analyzer (Zetasizer Nano-ZS; Malvern Instruments Ltd., Malvern, Worcestershire, UK). The device measured the electrophoretic mobility of the nanoparticles which is then transformed into zeta potential values contributed to prediction of material surface charges.

## 5. Conclusions

We try to explore the effect of polymer type on the fabrication of helical nanofibers Three polymer pairs involved in co-electrospinning experiments were studied. The experimental results showed that the Nomex/TPU system can fabricate helical nanofibers effectively via co-electrospinning. Based on the interfacial interaction induced by the polymer structure and intrinsic properties, we explore the effect of polymer type on the generation of helical structures from three aspects: polymer chain rigidity, miscibility and hydrogen bonding of the two solutions, and Coulomb force between the molecules and the solution surface. A larger rigidity differential of polymer chains of Nomex and TPU led to a larger interfacial interaction between them. At the same time, the hydrogen bonds between their polymer chains helped to obtain a partial miscible blend of the Nomex and TPU, and consequently increased the interfacial interaction between these two components. When the solutions were charged, an attractive force between the chloride ions contained in the Nomex molecules and the free charges on the solution surface was generated, leading to a longitudinal interfacial interaction in the Nomex/TPU system. This work provides an insight into the mechanism of helical fiber formation.
